# Supplementary material for: Effect of psychoeducation in reducing caregiver burden among family caregivers of people with mental disorders in Western Ethiopia, 2021–2022: A pre-test post-test study
Source: PLoS One. 2026 Apr 24;21(4):e0345260. doi: 10.1371/journal.pone.0345260 (PMC13108783; doi:10.1371/journal.pone.0345260)
Supplement: S2 Data — (DOCX) [file pone.0345260.s002.docx]

**Supplementary Material 1**

**Zarit Burden Interview (ZBI)**

**Instructions:** This questionnaire is designed to help us understand how you feel about your role as a caregiver. Please read each statement below and decide how often you feel that way. Then, for each item, please **circle the one number (0, 1, 2, 3, or 4)** that best reflects your experience.

**Scale for each item:**

**0** = Never

**1** = Rarely

**2** = Sometimes

**3** = Quite Frequently

**4** = Nearly Always

| # | Question | Never | Rarely | Sometimes | Quite Frequently | Nearly Always |
| --- | --- | --- | --- | --- | --- | --- |
| 1 | Do you feel that your relative asks for more help than he/she needs? | 0 | 1 | 2 | 3 | 4 |
| 2 | Do you feel that because of the time you spend with your relative that you don’t have enough time for yourself? | 0 | 1 | 2 | 3 | 4 |
| 3 | Do you feel stressed between caring for your relative and trying to meet other responsibilities? | 0 | 1 | 2 | 3 | 4 |
| 4 | Do you feel embarrassed over your relative’s behavior? | 0 | 1 | 2 | 3 | 4 |
| 5 | Do you feel angry when you are around your relative? | 0 | 1 | 2 | 3 | 4 |
| 6 | Do you feel that your relative affects your relationships with others in a negative way? | 0 | 1 | 2 | 3 | 4 |
| 7 | Are you afraid of what the future holds for your relative? | 0 | 1 | 2 | 3 | 4 |
| 8 | Do you feel your relative is dependent on you? | 0 | 1 | 2 | 3 | 4 |
| 9 | Do you feel strained when you are around your relative? | 0 | 1 | 2 | 3 | 4 |
| 10 | Do you feel your health has suffered because of your involvement with your relative? | 0 | 1 | 2 | 3 | 4 |
| 11 | Do you feel that you don’t have as much privacy as you would like? | 0 | 1 | 2 | 3 | 4 |
| 12 | Do you feel that your social life has suffered? | 0 | 1 | 2 | 3 | 4 |
| 13 | Do you feel uncomfortable about having friends over because of your relative? | 0 | 1 | 2 | 3 | 4 |
| 14 | Do you feel that your relative expects you to take care of him/her as if you were the only one? | 0 | 1 | 2 | 3 | 4 |
| 15 | Do you feel that you don't have enough money to care for your relative? | 0 | 1 | 2 | 3 | 4 |
| 16 | Do you feel that you will be unable to take care of your relative much longer? | 0 | 1 | 2 | 3 | 4 |
| 17 | Do you feel you have lost control of your life since your relative’s illness? | 0 | 1 | 2 | 3 | 4 |
| 18 | Do you wish you could leave the care of your relative to someone else? | 0 | 1 | 2 | 3 | 4 |
| 19 | Do you feel uncertain about what to do about your relative? | 0 | 1 | 2 | 3 | 4 |
| 20 | Do you feel you should be doing more for your relative? | 0 | 1 | 2 | 3 | 4 |
| 21 | Do you feel you could do a better job in caring for your relative? | 0 | 1 | 2 | 3 | 4 |
| 22 | **Overall,** how burdened do you feel in caring for your relative? | 0 | 1 | 2 | 3 | 4 |

**Beliefs Toward Mental Illness Scale (BMI Scale)**

**Instructions:** Please read each statement and circle the number that best reflects your opinion.

**Scale:**

**0 = Disagree**

**1 = Neutral / Undecided**

**2 = Agree**

| # | Statement | Disagree | Neutral | Agree |
| --- | --- | --- | --- | --- |
| 1 | People with mental illness are a danger to others. | 0 | 1 | 2 |
| 2 | People with mental illness are far less sociable than other people. | 0 | 1 | 2 |
| 3 | Mental illness is incurable. | 0 | 1 | 2 |
| 4 | Once you have a mental illness, you never really recover. | 0 | 1 | 2 |
| 5 | People with mental illness are a danger to themselves. | 0 | 1 | 2 |
| 6 | People with mental illness are difficult to talk to. | 0 | 1 | 2 |
| 7 | People with mental illness are unpredictable. | 0 | 1 | 2 |
| 8 | People with mental illness don’t tend to get along with others. | 0 | 1 | 2 |
| 9 | People with mental illness could “snap out of it” if they really wanted to. (R) | 0 | 1 | 2 |
| 10 | People with mental illness are hard to befriend. | 0 | 1 | 2 |
| 11 | People with mental illness will never be their normal selves again. | 0 | 1 | 2 |
| 12 | People with mental illness are irresponsible. | 0 | 1 | 2 |
| 13 | People with mental illness cannot have long-lasting friendships. | 0 | 1 | 2 |
| 14 | People with mental illness are unstable. | 0 | 1 | 2 |
| 15 | People with mental illness cannot handle responsibilities. | 0 | 1 | 2 |
| 16 | People with mental illness cannot “pull themselves together.” (R) | 0 | 1 | 2 |
| 17 | People with mental illness are unable to make their own decisions. | 0 | 1 | 2 |
| 18 | People with mental illness are best avoided. | 0 | 1 | 2 |
| 19 | People with mental illness are capable of maintaining a job. (R) | 0 | 1 | 2 |
| 20 | People with mental illness are incompetent. | 0 | 1 | 2 |
| 21 | People with mental illness are not capable of taking care of themselves. | 0 | 1 | 2 |

**(R) = Reverse-Scored Item.** For these items, scoring is inverted *after* data collection for analysis (Agree=0, Neutral=1, Disagree=2) so that a higher final score always indicates a less stigmatizing belief.
