# Supplementary material for: Effect of psychoeducation in reducing caregiver burden among family caregivers of people with mental disorders in Western Ethiopia, 2021–2022: A pre-test post-test study
Source: PLoS One. 2026 Apr 24;21(4):e0345260. doi: 10.1371/journal.pone.0345260 (PMC13108783; doi:10.1371/journal.pone.0345260)
Supplement: Attachment — Submitted filename: Point-by-Point Response to Editor and Reviewer Comments.docx [file pone.0345260.s004.docx]

**Point-by-Point Response to Editor and Reviewer Comments**

**Response to Editor Comments:**

We thank the Editor for the thorough review and for the opportunity to improve our manuscript. We sincerely appreciate the specific and constructive feedback provided. We have addressed each comment below and incorporated the necessary revisions into the revised manuscript.

1. **Title:**
   Thank you for this helpful suggestion. We have revised the title to include the year of data collection:
   *Effect of Psychoeducation in Reducing Caregiver Burden among Family Caregivers of People with Mental Disorders in Western Ethiopia: A Pre-Test Post-Test Study, 2021 - 2022.*
2. **Abstract – Study Setting:**

We appreciate this important clarification. We replaced "Western Ethiopia" with the specific administrative zones and towns:

"…was conducted at Nekemte Comprehensive Specialized Hospital in Nekemte Town, East Wollega Zone and Mettu Karl Comprehensive Specialized Hospital I Ilu Aba Bor Zone, Southwest Ethiopia."

1. **Abstract – Hospital Names:**

Thank you for raising this critical point. We had official permission letters from each hospital's administration stating the correct, official names.

**Abstract – Sample Size:**

We appreciate this valuable comment. We added a sentence in the abstract stating:
The sample size was determined using G*Power software, yielding a total of 556 family caregivers (intervention: n = 279; comparison: n = 277).

2. **Abstract – Study Participants:**

Thank you for highlighting the need for clarity. We clarified: The study participants were primary family caregivers of patients diagnosed with severe mental disorders (schizophrenia, bipolar disorder, or major depressive disorder). Caregivers in the intervention group received a structured family psychoeducation (FPE) intervention in addition to the routine clinical care (which included medication management and brief advice from treating psychiatrists)."

3. **Abstract – Protocol Registration:**

We appreciate this important methodological concern. The study protocol was not prospectively registered in a public trial registry. However, the full study proposal was reviewed and approved by the Institutional Review Board (IRB) of Jimma University. We indicated the IRB approval in the methods section and stated in the abstract: The study protocol was approved by the Jimma University IRB.

3. **Introduction – LMICs:**

Thank you for noting this terminology issue. We wrote it as *"low- and middle-income countries (LMICs)"* at first use.

1. **Introduction – FPE:** We appreciate this clarification request. We wrote it as *"Family Psychoeducation (FPE)"* at first use.
2. **Methods – Advantages for Participants:**

Thank you for this important ethical consideration. Participants did receive benefits, which we stated more clearly:

While no direct financial incentive was provided, participants in the intervention group received structured psychoeducation, which is a valuable supportive service not routinely available. All participants, in both groups, continued to receive standard psychiatric care for their patient-relative."

4. **Methods – Language:**

We appreciate this important contextual comment. We clarified: The intervention was delivered in Afaan Oromo, the local language spoken by all participants and the primary language of the study region, which ensured full comprehension."

1. **Methods – Intervention Room:**

Thank you for requesting this clarification. We added: The group sessions were conducted in a private, quiet meeting room within the outpatient department of each hospital, which was allocated by the hospital administration for the purpose of this study

5. **Results:**
   We appreciate this helpful observation. We performed a comprehensive English grammar edit of the entire Results section and the manuscript.
6. **Discussion:**
   We thank the Editor for this crucial and insightful feedback. We substantially rewrote the Discussion to:

(a) more deeply interpret our findings in the context of our clinical and academic experiences in Ethiopian mental health care;

(b) elaborate on the mechanisms (e.g., improved knowledge, coping skills, social support) that might explain the results, supported by relevant references; and

(c) move beyond simple comparison to synthesize why our findings aligned or differed from previous literature.

7. **Implications:**
   Thank you for this valuable suggestion. We expanded the "Implication" subsection to clearly distinguish between clinical practice (e.g., integrating FPE into routine care), policy (e.g., task-sharing and training non-specialists), and future research (e.g., longer-term follow-up, cost-effectiveness).
8. **General Grammar:**

We appreciate this overarching comment. As noted, we undertook a complete professional English language edit of the entire manuscript to ensure clarity and correctness.

**Response to Reviewer #2’s Comments:**

We extend our sincere gratitude for your thoughtful and detailed review of our manuscript. Your insightful comments were invaluable for strengthening our paper. We addressed each point below.

**Risk of Information Contamination:**

Thank you for raising this important methodological concern. To reduce information contamination, the intervention and comparison groups were recruited from different hospitals, ensuring that caregivers in the comparison group did not have access to or participate in the psychoeducation sessions during the study period.

1. **Study Population – Typo:**
2. Thank you for noting this error. We corrected "disorder" to "disorders."
3. **Exclusion/Inclusion Criteria:**

- **Duration of Care:** Thank you for requesting justification. We specified a minimum of six months to ensure caregivers had sustained experience of the caregiving role and clarified this rationale in the text.
- **One Caregiver per Patient:** We appreciate this methodological concern. This criterion ensured independent observations and prevented clustering effects, as the burden experience of multiple caregivers for one patient is not independent. We added this explanation.
- **Language (Afaan Oromo):** Thank you for highlighting this issue. The study was conducted in the Oromia region where Afaan Oromo is the predominant native language. This criterion ensured that participants could fully engage with the culturally and linguistically adapted intervention materials and group discussions without a language barrier. We made this rationale explicit.

4. **Repetition of Phrases:**

We appreciate this careful observation. We streamlined the criteria to avoid repetition, keeping the six-month duration only in the inclusion criteria section.

5. **Clarity on Registry and Patient Condition:**

- **Registry:** Thank you for seeking clarification. The outpatient psychiatric registry logged all patients attending follow-up. From this, we identified patients with the specified diagnoses and then approached their listed primary caregiver.
- **Patient Diagnosis:** We appreciate this conceptual comment. We focused on severe mental disorders (schizophrenia, bipolar disorder, major depressive disorder) precisely because caregiver burden is typically high and persistent in these conditions, making the intervention highly relevant. We stated this focus more clearly in the methods.

6. **Intervention Delivery without Incentives:**

Thank you for raising this practical concern. Participants were already attending the hospital for the patient’s routine monthly medication refills. The psychoeducation sessions were scheduled to coincide with these visits to minimize additional travel burden. We elaborated on this logistical planning in the methods.

7. **Data Analysis – Typo:**

We appreciate this careful review. We corrected "interaction" and all other typographical errors.

8. **Tense:**
   Thank you for pointing this out. We corrected the sentence to the past tense:
   "Study findings were shared with participants and the wider public through study reports and publication in an open access journal."
9. **Socio-demographic – Typo:**

Thank you for noting this error. We corrected "sex was" to "sex were."

10. **Discussion – Typo:**

We appreciate this correction. We corrected "Comparison."

11. **Conclusion – Typo:**

Thank you for identifying this issue. We corrected "disorders s" to "disorders."

12. **Source of Funding and Supervision:**

Thank you for raising this important transparency issue. The study was conducted as part of a PhD program. A mental health professional delivered the intervention under the regular supervision of the Principal Investigator (PhD student). No external funding was received for direct implementation costs (e.g., incentives), which was a study limitation. We added the following statement in the funding section: This research was conducted as part of a PhD thesis. No specific funding was received for the implementation of the intervention."

We sincerely thank both the Editor and Reviewer for their time, expertise, and constructive feedback. We believe these revisions have significantly enhanced the quality, clarity, and impact of our manuscript. We have submitted the revised version accordingly.
